# Coexistence of Systemic Lupus Erythematosus and Syringomyelia: A Case From a Rural Center in Nigeria

**DOI:** 10.7759/cureus.17679

**Published:** 2021-09-03

**Authors:** Airenakho Emorinken, Cyril O Erameh, Sylvester U Eluehike, Mercy O Dic-Ijiewere, Asuwemhe J Ugheoke

**Affiliations:** 1 Department of Internal Medicine, Irrua Specialist Teaching Hospital, Irrua, NGA; 2 Department of Radiology, Irrua Specialist Teaching Hospital, Irrua, NGA

**Keywords:** autoimmune disease, coexistence, cerebrospinal fluid, systemic lupus erythematosus, syringomyelia

## Abstract

Systemic lupus erythematosus and syringomyelia are two distinct conditions with different pathogenetic pathways as well as diverse genetic and clinical characteristics. The coexistence of these two conditions has not been previously documented in the literature. We describe a 38-year-old male who presented with progressive bilateral weakness and pain in the lower limbs and loss of sphincteric functions three years following a diagnosis of lupus nephritis. Relevant autoantibody testing, hypocomplementemia, and biopsy-proven membranous lupus nephritis confirmed the diagnosis of systemic lupus erythematosus and magnetic resonance imaging of the spine confirmed the presence of syringomyelia. Therapy for lupus nephritis was instituted accordingly, while the patient was referred for neurosurgical intervention. The mechanism underlying syrinx formation in this patient is uncertain and, thus, further research is critical in this area.

## Introduction

Systemic lupus erythematosus (SLE) is an autoimmune disease with a poorly understood etiology and varied clinical features. It follows a remitting and relapsing course and it is characterized by systemic inflammation and the presence of autoantibodies to nuclear and cytoplasmic antigens [1]. Syringomyelia is a descriptive term for any fluid-filled cavity within the spinal cord parenchyma that results from abnormal cerebrospinal fluid (CSF) circulation [2]. It is most frequently associated with Chiari 1 malformation, which accounts for half of the cases. Other causes include trauma, spinal cord tumors, post-inflammatory cord lesions, and secondary myelomalacia [3].

These two conditions are distinct in that they have separate etiopathogenic pathways as well as genetic and clinical characteristics. The coexistence of SLE and syringomyelia has not been previously reported in the literature. We describe a 38-year-old male who presented with progressive bilateral weakness and pain in the lower limbs and loss of sphincteric functions three years following a diagnosis of lupus nephritis.

## Case presentation

A 38-year-old Nigerian male presented three years ago (2018) with recurrent fever, oral ulcers, photosensitivity, non-scarring alopecia, and inflammatory polyarthritis involving the small joints of the hands, wrists, and elbow joints. He also had reduced urine volume and frothiness of urine. At that time, his test results were as follows: hemoglobin: 10.2 g/dL (12.0-18.5), leucocyte count: 2.04 ×10^9^/L (4-11), platelets: 250 × 10^9^/L (150-450), erythrocyte sedimentation rate (ESR): 106 mm/hr, and c-reactive protein (CRP): 3.2 mg/L (0-7.4). Urinalysis by dipstick revealed a protein of 3+ and hematuria. There was a positive antinuclear antibody (ANA) test with a titer of 1:640 (<1:80), anti-double-stranded DNA (anti-dsDNA): 54 IU/L (0-12), and an anti-Smith (anti-Sm) antibody. He had low levels of serum complement components: C3: 46 mg/dl (80-160), C4: 8mg/dL (15-57), and serum creatinine: 260 umol/L (57-113). Histopathologic analysis of his kidney biopsy revealed membranous glomerulonephritis, which corresponds to class V lupus nephritis.

He was then diagnosed as a case of SLE with nephritis (lupus nephritis) based on the presence of inflammatory arthritis, alopecia, oral ulcers, photosensitivity, anemia, leucopenia, raised ESR, positive autoantibody tests, hypocomplementemia, elevated serum creatinine, and biopsy-proven membranous glomerulonephritis. He was commenced on pulse methylprednisone at a dose of 1 gm/day and had a total of three doses, followed by oral moderate- to low-dose prednisolone. He was also given oral mycophenolate mofetil (MMF) at a dose of 1 gm twice daily for six months, then 500 mg twice daily, and Lisinopril at a dose of 10 mg daily. He responded favorably to treatment but was never seen again after eight months of follow-up until three years later.

In 2021, he presented to the accident and emergency department with a history of progressive bilateral weakness and pain in the lower limbs for two weeks and the inability to walk for four days. He had paraesthesia in the lower limbs and loss of sphincteric functions (bladder and bowel). There was no difficulty swallowing, diarrhea, myalgia, headache, nausea and vomiting, no blurred vision, and fever. No symptoms of the respiratory system were noted and the upper limbs were spared. There was no history of trauma, recurrent low back pain, or history of previous bladder outlet obstruction. There was no medical history of diabetes mellitus or hypertension. At this presentation, symptoms of oliguria, inflammatory arthritis, alopecia, and oral ulcers were absent. The patient, however, was still regular on MMF.

He had a temperature of 36.8°C, 98 percent oxygen saturation in room air, a pulse rate of 86 bpm, a respiratory rate of 18 breaths per min, and blood pressure of 120/80 mmHg. A neurological examination revealed normal higher cerebral function and cranial nerves. His global muscle strength in both lower limbs was grade zero (0) on the Medical Research Council Manual Muscle Testing Scale. He had reduced tone and the deep tendon reflexes of the knees and ankles bilaterally were absent. There was impaired pain and temperature sensation, two-point discrimination, joint position, and vibration sense in both lower limbs. He had a sensory level of about T10 dermatomes. The bilateral Babinski test (plantar) reflexes were flexor. He had a loss of anal sphincteric tone on the digital rectal examination. The examination of the upper limbs, cardiovascular, and respiratory systems were normal. A provisional diagnosis of acute transverse myelitis secondary to SLE was made.

**Figure 1:**
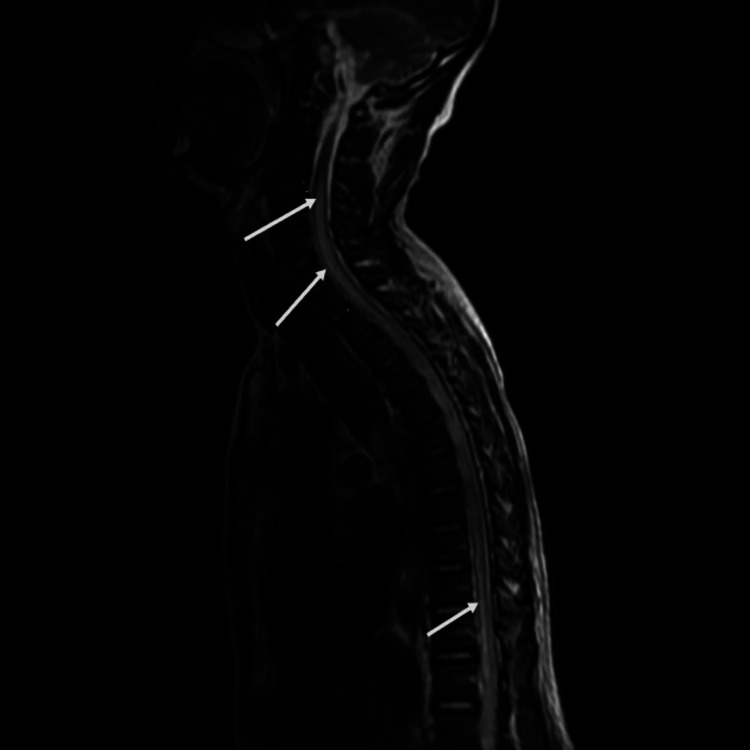
Sagittal section of T2-weighted MRI showing a marked hypertense long segment dilatation of the central canal extending from the lower border of the C3 vertebrae throughout its distal length with associated peripheral thinning of the cord parenchyma.

**Figure 2:**
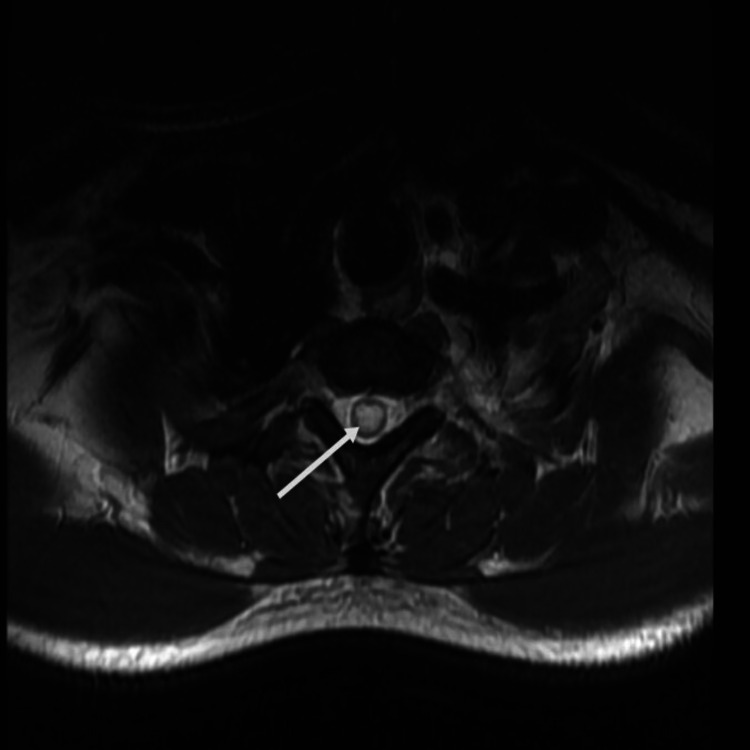
Axial section of T2-weighted MRI showing a central circular hyperintensity within the central canal.

His investigation results were as follows: ESR: 60 mm/hr, CRP: 2.1 mg/L (0-7.4), leucocyte count: 3.45 ×10^9^/L, hemoglobin: 13.6 g/dL, serum creatinine: 110 umol/L, ANA: 1: 640 (< 1:80), and positive anti-ds-DNA and anti-Sm Ab. He had low levels of serum complement components: C3: 50 mg/dL and C4: 10 mg/dL. Anti-Ro (SSA), anti-La (SSB), antiphospholipid antibodies, thyroid function test (TFT), vitamin B12 assay, and serum electrophoresis were normal. Serologic tests were negative for syphilis, HIV, and hepatitis B and C. The Mantoux test was 0 mm; urinalysis, serum prostate-specific antigen, and total serum protein were all normal. The cerebrospinal fluid analysis was unremarkable with no albuminocytological dissociation. The patient was evaluated with an MRI of the spine, which revealed an expansile intramedullary T2-weighted hyperintense signal intensity within its central canal extending from the lower border of the C3 vertebrae level extending throughout its distal length (Figures 1, 2). This confirms the diagnosis of syringomyelia coexisting with SLE previously diagnosed.

Nerve conduction studies and electromyography were not done. The patient was to continue follow-up for lupus nephritis as initially prescribed and was referred to a neurosurgeon for possible surgical intervention. However, this was yet to be done at the time of this report.

## Discussion

The coexistence of SLE and syringomyelia has not been previously documented in the literature. This case presentation suggests such a possibility. The only similar case documented in literature was the development of a syrinx in a patient with an inflammatory central nervous system (CNS) disease “suspected” to be SLE [4]. In this report, our patient had both clinical and serologic confirmation of SLE and imaging evidence of syringomyelia. The diagnosis of SLE preceded that of syringomyelia in this patient by two years. While SLE is a predominantly female disease, syringomyelia has no gender predilection [5]. Both disease entities are found commonly in the third and fourth decades of life [5], and our patients fall within this age range.

Nervous system damage is an important cause of mortality in patients with SLE [6]. The most prevalent CNS manifestation is intractable headache, which accounts for more than half of all cases [7]. Others are cerebrovascular accidents, seizures, depression, and psychosis, aseptic meningitis, demyelinating syndromes including transverse myelitis, Guillain-Barre syndrome, and optic neuritis, myasthenia gravis, movement disorders, and neuropathies [8]. The exact pathogenic etiology of neuropsychiatric SLE is not clear but could be multifactorial involving genetic, immunological, and environmental factors [8,9]. Several inflammatory cytokines and pathogenic autoantibodies have been implicated. Pathologic CNS findings include thrombotic multifocal infarcts of the small intracranial vessels, endothelial proliferation, and perivascular inflammation [8,9]. There have also been reports of SLE coexisting with multiple sclerosis, though rarely [10].

The pathophysiology of syringomyelia is uncertain. However, several hypothetical theories have been postulated to explain the development of syringomyelia [3,11]. Syringomyelia has been associated with non-infectious inflammatory lesions [12]. Hypotheses on the pathophysiology of non-infectious inflammation-associated syringomyelia include coincidence, necrosis, or ex-vacuum central canal dilation due to myelin/axonal damage [4]. The clinical course progresses over months to years with an early rapid deterioration that wanes over time [13]. Although there are no specific symptoms associated with syringomyelia, sensory abnormalities, motor weakness, and pain are the most common manifestations [3]. Our patient similarly presented with these manifestations and had no previous history of trauma or an infectious illness.

Antiphospholipid syndrome (APLS) can cause thrombosis of several vessels, including those supplying the spinal cord [14,15]. Although spinal cord infarction from APLS is very rare, there have been some case reports [15]. This may arise from thrombosis of arteries branching from the artery of Adamkiewicz, most commonly the anterior spinal artery that supplies the lower two-thirds of the spinal cord [15]. However, the most frequent cause is surgical repair of the thoracoabdominal aortic aneurysm. The clinical presentation includes sudden onset paralysis, bladder/bowel dysfunction, and the presence of a sensory level. The MRI findings of focal cord swelling, “pencil-like,” and “owl-eyes” sign hyperintensities on T2-weighted images aid diagnosis [16]. The index case did not have an inciting event, was negative for antiphospholipid antibodies, and had no evidence of infarction on MRI.

The coexistence of SLE and syringomyelia in this patient may be coincidental, as syringomyelia is becoming more prevalent and can be discovered incidentally as a result of the increased use of MRI in the routine evaluation of back and neck pain [12]. It is possible that the autoimmune inflammation associated with SLE contributes to the formation of syrinxes. Inflammatory cord lesions such as transverse myelitis can trigger the development of syringomyelia. The edema associated with inflammatory cord lesions may disrupt CSF hydrodynamics, resulting in decreased CSF circulation and enlargement of the central canal [17,18]. Additionally, post-inflammatory fibrosis decreases the compliance of the spinal meninges, impairing CSF hydrodynamics.

Despite the fact that our patient's ESR was elevated, indicating an inflammatory process, the CSF did not contain an increased number of inflammatory cells and the MRI showed no evidence of myelitis. The largely unclear pathogenic pathways of both conditions also make it difficult to determine whether there is a causal or contemporaneous relationship between them or if it is simply coincidental. There is no epidemiologic data on the risk of SLE and syringomyelia coexisting. Therefore, the question of whether SLE is causally linked to syringomyelia is one that cannot be answered definitively based on the available information. As a result, further research may be necessary in this regard.

## Conclusions

In conclusion, it is not established if there is a causal relationship between SLE and syringomyelia or if it is just a coincidence. This is because the pathogenic mechanism underlying syrinx formation is still largely unclear. Possible mechanisms include CSF hydrodynamic disturbances and inflammatory and/or ischemic necrosis. Further research in this area may be crucial and should focus on prospective studies.
